# Changing Paradigms of Bedside Clinical Teaching

**DOI:** 10.7759/cureus.8099

**Published:** 2020-05-13

**Authors:** Iqbal Ratnani, Sahar Fatima, Anam Mithwani, Justyn Mahanger, Zehra Surani

**Affiliations:** 1 Critical Care Medicine, Debakey Heart and Vascular Center, Houston, USA; 2 Critical Care, Houston Methodist Hospital, Houston, USA; 3 Education, University of Toronto, Toronto, CAN; 4 Humanities, York University, Toronto, CAN; 5 Medical Education and Simulation, It's Your Life Foundation, Corpus Christi, USA

**Keywords:** medical education, learning theories, bedside teaching, millennials, gogies, collaborative learning

## Abstract

Education has continued to evolve since the existence of mankind. We have now become more well-equipped and refined our teaching methodology over time. Despite all the progress made, we still continue to rely on bedside huddle for teaching. Globalization has facilitated learning in culturally diverse environment. Grand rounds, noon lectures and conferences help the millennial house-staff to effectively translate their concepts to hands-on clinical experience. Useful teaching strategies like story-telling, connecting, simplifying, playing hierarchy make learning more fun and engaging. Multidisciplinary collaborative learning has eased conceptualization and retention of practical knowledge. Our education system now focuses more on patient-centered, case-based learning system.

## Introduction and background

Learning has existed since the beginning of humanity. The human curiosity continues to explore new avenues of knowledge. Moreover, humans have this innate desire to transfer knowledge to others. A famous educationist once said, “The ability to learn is not what makes us human; it is the ability to teach” [1]. Patient’s bedside is a diverse platform where the participants actively engage, learn and acquire new skills. It is the first in-person professional development learning environment for house staff and fellows and an opportunity to practice hands-on clinical medicine.

## Review

**Figure 1:**
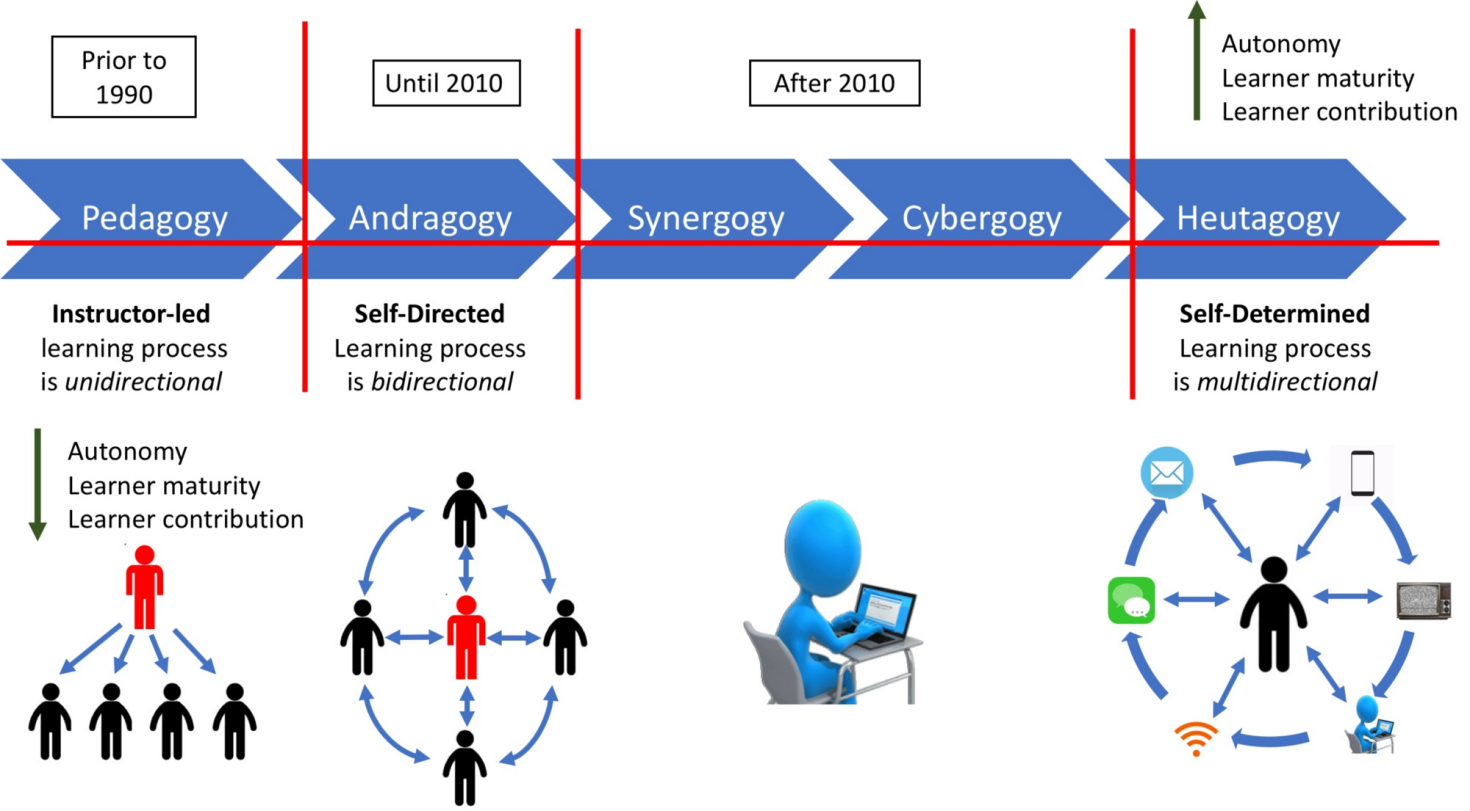
Learning theories at a glance

As humans evolve through various cultures, geographies, catastrophes, religious and political revolutions, different learning theories continue to form. As tools of imparting knowledge expanded, marked by psychology, culture and previous experiences, people realized that every person has his own unique style of learning, and more and more learning theories came into being [2,3]. As the science and art of knowledge got more refined and organized, many of these theories got tested with measures of outcomes, leading to the formation of more theories. Even though classically there are three fundamental learning theories, i.e., behaviorism, cognitivism, and constructivism, new concepts of ‘gogies’ including but not limited to pedagogy, heutagogy, peeragogy and andragogy continue to be added [4]. With the advent of the internet and rapid globalization, other terms such as cybergogy are being introduced, and we expect to hear many such new terminologies in the future (Figure 1) [5].

Human psychology attains experiences, and it continues to change one’s own worldview through perpetual new experiences integrating with prior experiences. In other words, we evolve through our experiences. The intelligent and curious human mind continues to reflect, reconcile, discard, question, assess, and explore old information through the discovery of new information. Bruner coined this constructivism with term ‘spiral learning’ [6].

At the educational level in general and bedside clinical teaching in particular, the concept of spiral learning is reinforced. It makes learning active, engaging, and creative. It enhances the thinking capabilities, social and communication skills, and a sense of achievement by the house-staff. Although traditionally, formal classroom lectures, grand rounds, and mini-conferences stay the core method of teaching, heutagogy i.e., active participation from a learner is the new standard [7]. Allowing students to have maximum hands-on participation at the patient’s bedside, and to have an un-intimidating discussion with teaching attending as well as with peers is the key to the success. Metacognition at the level of residency and fellowship training can be trusted with house-staff taking responsibility for their own learning [8].

Globalization of healthcare and its effect on learning

As the world is getting globalized and house-staff from diverse cultures are coming together “learning in a social context” is a relatively new concept and will require more emphasis in coming years. The concept of globalization is not new to mankind, its roots can be traced back to the 15th and 16th century exploration voyages by Columbus and Magellan. The first global initiative in healthcare occurred in the 6th and 7th century when Tibetan Empire, King Songtsen Gampo (605-650) organized the first international medical conference. Invitations were extended to physicians from South-east Asia [9]. This initiative opened up gateways for future collaborations and hence laid the foundations of new “culture” getting formed globally, which is now being fueled by social media collaborations. It would not be surprising if in coming years, the notion of ‘checkmarks’, fulfilling the requirement of rotations get considered as barriers, and “dynamic active learning” take new forms like distantly connected team-works, and group projects, and ‘multi-institutional-student-centered’ learning. Millennial students and generation z (refers to demographic cohort of people born in late 90’s and early 21st century) are special, sheltered, and pressured, but at the same time, they are often more team-oriented, confident, and achieving [10,11]. Online collaboration is growing rapidly at an exponential rate. It is necessary to utilize this modem in the most efficient way possible, simultaneously keeping the credibility of knowledge exchange. In future, the house-staff will be required to be skilled in “information management”, an art of two-way communication which will be followed by meaningful feedback from supervising teaching attendings.

Pitfall of prior knowledge

By default, all house-staff bring prior knowledge (PK) to the bedside. Some of this PK can be determined objectively while some cannot as it is more embedded in psychology and based on prior life experiences and is technically called a schema or cognitive psychology [12]. House-staff come to bedside relatively polished after finishing four years of medical school and, in some cases, three years of residency (fellows). Most of them have a solid understanding of human physiology and anatomy, but frequently also carry a distorted understanding of the pathology of various diseases, and this is the reason that Pathology Competencies for Medical Education (PCME) model is being incorporated into the medical education system [13]. Inappropriate PK can hinder present learning. One classic example of this can be seen through patients who arrive at the cardiothoracic intensive care unit (ICU) with acute aortic dissection. Pre-operatively, low blood pressure and a low heart rate is desired to decrease the pressure on the dissecting aorta. On the contrary, post-operatively, many of these patients may require higher blood pressure to avoid spinal ischemia. This is extremely critical since having postoperative low blood pressure for only a few hours can render a patient to permanent paraplegia, and treatment should be guided by a pressure difference between mean arterial pressure and spinal pressure [14]. House-staff, if unaware of this contrast in management can expose their patients to potentially life-threatening consequences . In this case, house-staff needs to be guided by their prior knowledge of arterial supply of spinal cord. As the artery of Adamkiewicz is a single vital arterial supply to the spinal cord therefore the artery and its collateral need to be kept open via relatively high pressure after a thoracic endovascular aortic aneurysm repair [15]. Another example is the common knowledge and traditional teaching in medical schools that beta-blockers (BB) should not be administered to patients with asthma. However, in ICUs a very short-acting type of BB (esmolol) can be used as an intravenous infusion to control high heart rates and blood pressure very safely, even in patients with high broncho-reactivity [16]. This stereotyped PK regarding BB is so resistant that it requires multiple explanations to students via different methods of teaching for them to understand, including explicitly linking new (research) material to PK, applicability of different classes of BB, showing where and why analogies are breaking down, discussing their reasoning, and above all, allowing time for house-staff themselves to experience many times the safety of esmolol in asthmatic patients who arrive with high ventricular rates of atrial fibrillation [17]. Doing pre-assessments by having a formal test prior to rotation is an effective way to gauge PK in order to help guide with what level of teaching needs to be pursued [18]. Also, asking them to draw concept-mapping could be a productive way of assessing PK [19].

‘Superficial’ to ‘conceptual’ learning

**Figure 2:**
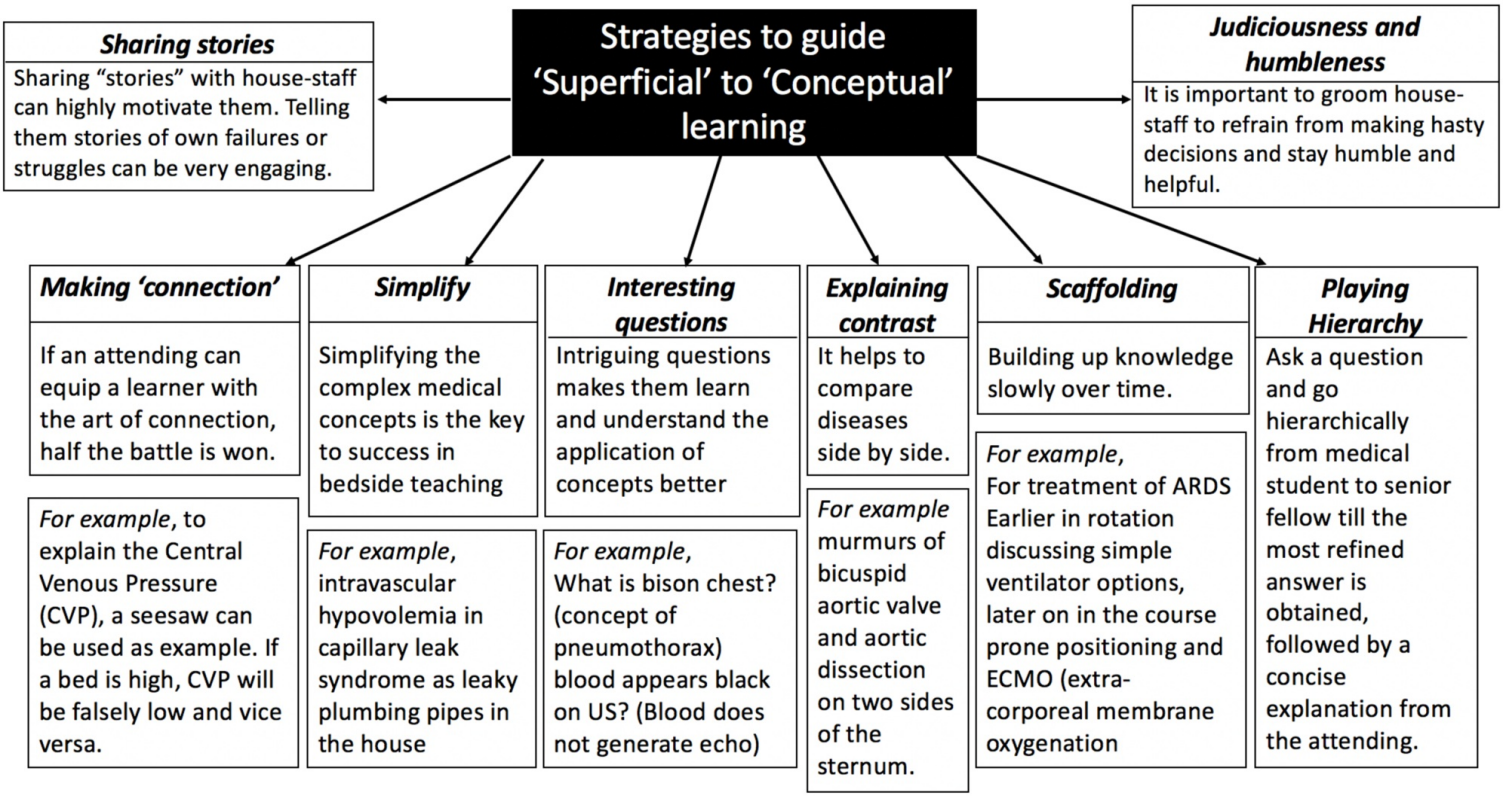
Strategies to guide ‘Superficial’ to ‘Conceptual’ learning

This kind of learning occurs when the house-staff develop the ability to think out of the box, establishing the connection between bookish facts implemented with its twists in medical practice. Teaching attending plays a pivotal role to guide the house-staff from surface to deep learning. Few strategies can be very useful (Figure 2).

It is important to teach them to cerebrate on all workup and developments, considering views of all consultants and taking time in deciding further plan is important. It is essential to stay away from the trap of AMS (Already Mastered Syndrome) and to avoid to make statements like “I am very good at performing arterial lines”, “I already learned tricks on intubating obese patients” or “it is not hard to put a chest tube.” Frequent reminders should be sent that any procedure can turn into a nightmare if they are not adequately prepared and a support system exists to back them up in time of crisis. Simultaneously, it is important to teach students when to restrain from throwing the whole kitchen sink into management of the patient. It is vital to focus on teaching them the judicious use of arsenals in their weaponry.

Getting personal and collaborative learning

The hospitals are relatively tense environment, and most of the house-staff enter the rotation with some apprehension and a sense of fragility. Day 1 of rotation is crucial to open on a positive note with a welcoming attitude, trying to be personal by getting to know their first names and even providing cell phone numbers so they don’t feel in a vacuum bearing a sense of hopelessness. On day one, while giving them orientation, it is important to explicitly articulate expectations, identify challenges, provide rubrics, and have a dialogue so team can work together, align mutual objectives, and enhance efficacy. House-staff come to any rotation not only to have a good exit evaluation as an attainment value but also to genuinely feel comfortable managing sick patients in order to fulfill their intrinsic value. Teaching attending rather than just supervising, needs to work side by side while discussing their decisions in clinical management. If house-staffs feel valued and respected, they graduate from their rotations with high motivation. “Collaborative learning” is most applicable in medical teaching environment [20]. When a group of residents feel teamed together and having social interaction with their attending, their learning efficacy synergistically goes up. Also, the positive interdependence between residents comes into play. Who will pick the next admission? Who will perform the procedure? How accurate the daily progress note should be for the covering residents? And who will track the flow of the day? All become automatic building blocks of collaborative learning. Team-based learning (TBL), scaffolding, and cognitive co-construction come into play with shared knowledge without one person being under the spotlight so not to feel to have a cognitive load. Case discussions at the patient’s bedside enhance problem-based learning (PBL). These interactions lead to improved self-efficacies, confidence, Self-Directed Learning Readiness (SDLR) and a goal-directed behavior [21]. Self Determination Theory (SDT) plays an underlying role in collaborative teaching to attain residents’ intrinsic motivation [22]. This can be enhanced by providing relevant articles, and giving residents the opportunity to speak and reflect - a mode of asynchronized teaching [23]. This can be challenging whenever there is an avoidant student [24]. They can be motivated by engagement and achievement. Overall goal of collaborative learning is to steer house-staff from the stage of unconscious incompetence to the stage of conscious or unconscious competence by encouraging reading, feedback, prompting, concept building, group discussions, emphasizing keywords and frequently going back to the learned concepts. Small group tutorials with “inquiry processes” can also be a valuable way of teaching [25].

Feedback

Human physiology is complex, and the understanding of disease pathology requires a lot of conceptual understanding. This requires medical students and residents to do a lot of “elaboration”. But “feedback” is considered a cornerstone of clinical teaching. Going over house-staff admission/physical of a patient, daily progress notes, and supervising them for procedures are all grounded on the basic principle of “feedback.” Effective feedback can be strategized in various forms such as schematizing, summarizing, rephrasing, or applying information to another situation. No matter how advanced or different the educational modes become, elaboration and feedback from teaching attending will always remain a key principle of educational learning and will continuously ride on the inner spark of a learner’s motivation.

As students are getting more and more constraint with ever-expanding knowledge horizons, teaching the skill of ‘time management’ and “do more with less” would become pivotal. Despite all the costly gadgets, there are some basics that never change - the only requirement being the creativity of the teacher and the enthusiasm of the learner. An example is to draw simple doodles to teach difficult concepts. Use of YouTube videos, Google images, and computer-based modalities work rapidly for the millennial generation, who connect with multimedia easily for information [15,26].

In short, feedback is more of an art than a methodology to balance strengths and weaknesses.

Diversity of house-staff sub-specialties

It is interesting, and even enjoyable, when residents from different sub-specialties rotate together, as they all approach a given patient very differently. Residents from surgical and anesthesia are prone to jump to procedures like intubation or placement of central venous or arterial lines, whereas residents from an internal medicine may step back and brainstorm the management strategy before initiating any treatment. Teaching attending may need to find a common ground to explain that both procedures and management plans need to happen simultaneously as time is critical from both procedure as well as disease-management perspectives. One can’t take precedence over others. This is a classic example of limitations of analogy that need to be guided by heuristics to avoid inappropriate application of knowledge, and explicitly identify the discipline-specific conventions [17]. One of the key aspects for a teaching attending to remember is the fact that students come in all shapes and sizes! If an instructor is not willing to be flexible to accommodate the needs of each student, he may not be successful in his teaching methods despite being an expert in his field.

Learning climate

It needs to be remembered that house-staff are usually young and still going through the different various stages of social development. Any insensitive comment or change in tone can covertly or overtly marginalize a member in the team, particularly since US healthcare teaching system receives a large number of international students [27,28].

Holistic and healer approach

It is essential to teach house-staff holistically as much as possible and not only the contents of the curriculum, and honesty with their profession as well [29]. It is important to establish a mindset of a healer rather than simply being a manager of the diseases. Encouraging them to go through their rotation with ‘flow’ keeps them satisfied as a learner and their rotation becomes engaged and meaningful [30,31]. It is extremely important to equip them with tools to be responsible decision makers and be compassionate (to patients and families) [32]. This is the attending’s responsibility that house-staff are given the opportunity to find the meaningful horizon of the practice of medicine [33]. Values congruence can be a profound predictor of one’s teaching [34]. Comprehensive approach of teaching described as SEEAE (social, emotional, ethical, and academic education) by Cohen can be a guiding manual in this regard [29].

## Conclusions

Due to lessons learnt from the past and global digitalization, teaching methodology has continued to evolve, improve and intercept human brain learning mechanics. The merger of old-school, hard-core theoretical model with the newer, well improvised bedside clinical teaching has revolutionized the education system. The newer model facilitates the concept of ‘growing and learning together’ in comparison to its predecessor unidirectional educational flow system from teacher to student. Modern-day house staff and fellows feel more confidant and well-equipped to face challenges in the practical world.
